# [Retracted] MicroRNA-181a regulates epithelial-mesenchymal transition by targeting PTEN in drug-resistant lung adenocarcinoma cells

**DOI:** 10.3892/ijo.2025.5784

**Published:** 2025-08-05

**Authors:** Haihui Li, Pei Zhang, Xiaojin Sun, Yiming Sun, Chao Shi, Hao Liu, Xuegang Liu

Int J Oncol 47: 1379-1392, 2015; DOI: 10.3892/ijo.2015.3144

Following the publication of the above paper, it was drawn to the Editor's attention by a concerned reader that, regarding the scratch wound and cell migration and invasion assay experiments shown in Fig. 2A and B and Fig. 5A-C, a large number of data panels showed evidence of overlapping data, both within the same figure parts and comparing between figures. Similarly, evidence was also uncovered of data duplication comparing the cell microscopic images in Figs. 6A, 7B and 8B.

Owing to the large number of data duplication events that have been identified in this paper, the Editor of *International Journal of Oncology* has decided that it should be retracted from the Journal on account of a lack of confidence in the presented data. The authors were asked for an explanation to account for these concerns, but the Editorial Office did not receive a reply. The Editor apologizes to the readership for any inconvenience caused.

